# Tapinarof Nanogels as a Promising Therapeutic Approach

**DOI:** 10.3390/pharmaceutics17060731

**Published:** 2025-06-01

**Authors:** Barbara Balogh, Ágota Pető, Pálma Fehér, Zoltán Ujhelyi, Ildikó Bácskay

**Affiliations:** 1 Department of Pharmaceutical Technology, Faculty of Pharmacy, University of Debrecen, Rex Ferenc Utca 1, H-4002 Debrecen, Hungary; balogh.barbara@pharm.unideb.hu (B.B.); feher.palma@pharm.unideb.hu (P.F.); 2 Doctoral School of Pharmaceutical Sciences, University of Debrecen, Nagyerdei Körút 98, H-4032 Debrecen, Hungary; 3 Department of Industrial Pharmaceutical Technology, Faculty of Pharmacy, University of Debrecen, Rex Ferenc Utca 1, H-4002 Debrecen, Hungary; ujhelyi.zoltan@pharm.unideb.hu; 4 Institute of Healthcare Industry, University of Debrecen, Rex Ferenc Utca 1, H-4002 Debrecen, Hungary

**Keywords:** tapinarof, non-steroid, aryl hydrocarbon receptor, dermatological therapies, nanotechnology for psoriasis

## Abstract

Psoriasis is a chronic inflammatory skin disease characterised by increased oxidative stress, the overproliferation of keratinocytes, the accumulation of inflammatory mediators, and skin barrier damage. Although a number of therapeutic options are available, finding long-term treatments that are well-tolerated and patient-friendly treatments remains a challenge. Tapinarof is a new type of aryl hydrocarbon receptor (AhR) modulator that has recently attracted attention as a promising non-steroidal alternative. However, its application may be limited by its poor water solubility and low degree of skin penetration. Nanotechnology-based drug carriers, specially nanogels, offer new opportunities to overcome these limitations by combining the advantages of targeted drug delivery and enhanced skin penetration. Furthermore, nanogel formulations can improve skin hydration and support the restoration of skin barrier function, which are important in the treatment of psoriasis. This review focuses on current and emerging therapeutic approaches, with particular emphasis on the potential of incorporating tapinarof into nanogel formulations as a novel alternative to topical psoriasis treatment.

## 1. Introduction

Although several reviews have already discussed the pathophysiology and treatment of psoriasis, most have focused on either systemic biological therapies or conventional topical treatments. However, a significant number of patients with psoriasis still try to avoid the use of biologic agents and corticosteroids, and there is still a strong need for well-tolerated topical treatments that can be used in the long term. Despite this, few publications have explored the formulation-specific challenges and opportunities associated with newer topical agents.

Tapinarof is a recently approved topical aryl hydrocarbon receptor (AhR) agonist that has emerged as a promising therapeutic option due to its anti-inflammatory, antioxidant, and barrier-restoring properties. However, despite its growing clinical relevance, there is a lack of comprehensive discussion on how its efficacy and safety could be further optimised. This review aims to bridge that gap by combining current knowledge on tapinarof with pharmaceutical strategies designed to enhance its performance.

Among these strategies, nanogels deserve particular attention for their ability to improve skin penetration, offer controlled drug release, and reduce systemic side effects, properties that are beneficial for chronic inflammatory skin diseases such as psoriasis. While nanotechnology-based drug delivery systems are increasingly being explored in dermatology, the incorporation of tapinarof into nanogel formulations is a novel and unexplored area. This review summarises current progress and emerging trends, highlighting the potential of advanced carrier platforms to improve the therapeutic profile of tapinarof. This review also offers a unique perspective to help readers better understand where emerging therapies, such as nanogel-based approaches, fit into the current context of psoriasis treatment.

## 2. Methodology

This review was conducted using a structured search of peer-reviewed, English-language scientific articles, clinical trial reports, and regulatory documents related to psoriasis, tapinarof, and nanogel-based drug delivery systems. Only publications from 2014 to 2025 that were written in English were considered. Databases searched included PubMed, Scopus, Web of Science, PubPharm, and Google Scholar including the following terms: “tapinarof”, “nanogel”, “psoriasis”, “topical treatment”, “AhR agonist”, “drug delivery systems”, “nanotechnology in psoriasis”, “dermal drug delivery”, and “tapinarof in dermatology”. In addition, clinical trials related to tapinarof were identified through www.clinicaltrials.gov (accessed on 9 March 2025). Articles were selected based on their relevance to the topic of the review, with priority given to recent studies and those involving regulatory approvals, particularly by the FDA.

## 3. Structure and Barrier Function of the Epidermis

The skin is the largest organ in the human body, operating as a multifunctional defence system and maintaining homeostasis. The structure of the skin is extremely complex and divided into three major layers: the epidermis, the dermis, and the hypodermis. Each has different anatomical and functional characteristics [1,2,3].

The epidermis is composed of five different layers: the uppermost layer is the stratum corneum, followed by the stratum lucidum, stratum granulosum, stratum spinosum, and stratum basale. As the epidermis is the most external layer of the skin, it consists of exfoliating epithelial cells, called keratinocyte cells, that are constantly renewing themselves, ensuring the integrity of the outer protective layer. When exposed to UV radiation, the keratinocytes of the epidermis synthesise vitamin D, which is essential for calcium metabolism and the proper functioning of the immune system. It also contains specific cell types such as melanocytes, Langerhans cells, and Merkel cells, which are responsible for pigmentation, immune defence, and sensory function. In addition, the sebaceous and sweat glands of the skin perform exocrine functions, helping to keep the skin hydrated and eliminating toxins. The functions of the sweat glands is also crucial for thermoregulation, as they reduce body temperature by evaporating sweat. The stratum corneum prevents water loss and the penetration of pathogens due to the presence of a hydrophobic lipid layer [1,2].

Overall, the upper layer of the skin not only provides physical and chemical protection, but it is also an active participant in the metabolic processes of the body [2].

## 4. General Characteristics of Psoriasis

Psoriasis is a chronic immune-mediated skin disease that affects 1–3% of the world’s population, more than 100 million people [4,5,6,7]. It can emerge at any age, but it typically first appears between the ages of 15 and 25, with a second peak in emergence observed between the ages of 50 and 69 [8,9].

Genetic, environmental, and immunopathological factors all influence the pathogenesis of this disease [6,9,10,11]. Familial accumulation and specific gene variants (e.g., genes involved in the regulation of immune response and keratinocyte proliferation) may also contribute to its development. The initial presentation of psoriasis and subsequent flare-ups can be provoked by a variety of environmental factors, such as infections, stress, smoking, alcohol consumption, and cold, dry weather [1,6,9].

Both the adaptive and innate immune systems take part in the pathogenesis of psoriasis, characterised by a mixture of autoimmune and autoinflammatory mechanisms, with a central role played by antigen-presenting cells (APCs) and inflammatory cytokines (TNF-α, IL-17, and IL-23) [6,9,11,12,13,14]. These inflammatory responses result in keratinocyte hyperproliferation, which lead to a thickening of the skin and the typical plaque lesions [1,14,15].

Psoriasis is not a contagious illness, but it is associated with significant inflammation that manifests as painful, itchy, scaly plaques that show intermittent remission and exacerbation [16,17,18,19]. Skin cells normally mature and slough off in about a month, whereas in psoriasis this process takes only 3–5 days, leading to an accumulation of cells [20].

There is currently no permanent cure, but symptoms can be controlled and the quality of life can be improved with the right therapy [8,19]. The National Psoriasis Foundation categorises psoriasis as mild (<3%), moderate (3–10%), and severe (>10%) according to the extent of the disease and the proportion of the affected body surface. The severity of psoriasis is measured by calculating a PASI (Psoriasis Area and Severity Index) score ranging from 1 to 10, where 10 indicates severe and 1 indicates good psoriatic skin condition. [6].

Psoriasis can occur in several forms, which can be classified into the following types according to the American Academy of Dermatology [18]:

- Plaque psoriasis (psoriasis vulgaris): The most common form (>80% of cases), associated with erythematous plaques covered with silvery scales [18,21,22]. This usually appears in the elbows, knees, scalp, navel, and sacrum regions [19,23].
- Guttate psoriasis: Appears mainly in children and young adults, with drop-shaped erythematous lesions, often after infections [8,23,24].
- Pustular psoriasis: A less common but more severe form with sterile, pustular blisters, which can be localised (palms, soles) or generalised (involving the whole body surface) [23,24,25].
- Erythrodermic psoriasis: The most severe form, affecting more than 90% of the skin and associated with severe inflammation, scaling, oedema, and even life-threatening complications [23].
- Intertriginous psoriasis (inverse psoriasis): A form that appears in the folds of the body (armpits, groin) and is associated with red, scaleless, painful plaques [8,23,25].

Psoriasis not only damages the skin but it can also trigger inflammatory processes throughout the body, which can be associated with a number of comorbidities, affecting the daily lives of patients [21,26]. These inflammatory processes can contribute to cardiovascular disease, metabolic syndrome, obesity, and inflammatory bowel disease [6,19,21]. In addition, psoriatic arthritis evolves in 10–30% of cases and manifests as arthritis. It can lead to the erosion of cartilage tissue in joints and cause irreversible joint damage in the long term [12,18,19,21]. Psoriasis also increases the risk of depression and anxiety threefold, which can be a consequence of the social stigmatisation and chronic pain associated with skin symptoms [22,27]. In the early-onset form of the disease, around 75% of patients experience more severe comorbidities, while the late-onset form has a higher prevalence of other types of comorbidities [19]. Although the disease has a fluctuating progression and it is currently incurable, targeted therapies can provide significant improvements in quality of life [8].

## 5. Immunological and Molecular Pathogenesis of Psoriasis

The most important of the pathogenesis is uncontrolled proliferation of keratino-cytes and dysregulation of the immune system that lead to abnormal skin lesions [16,27,28]. The condition of this disease becomes autoimmune as the cells of the body activate the immune system [24].

**Figure 1:**
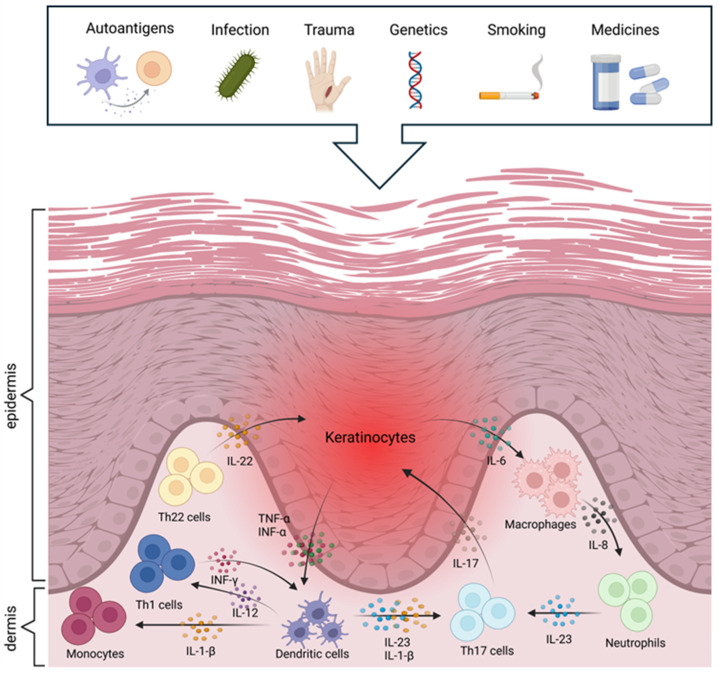
Aetiology and pathomechanisms of psoriasis (created with BioRender.com).

The evolution of psoriasis can be described in two main phases: initiation and maintenance. In the initiation phase, various environmental factors, such as stress, trauma (e.g., tattooing), bacterial or viral infections (e.g., streptococcal pharyngitis in guttate psoriasis), drugs (e.g., beta-blockers, lithium), and dry, cold weather conditions, can lead to the activation of the disease [10,19,21,27]. In the maintenance phase, chronic activation of the immune system, overproduction of inflammatory mediators, and abnormal cell proliferation ensure the persistence of the condition. Several components of the immune system active participate in the maintenance of the disease [21]. Dendritic cells, T cells (particularly Th17 cells), and various other immune cells, including neutrophils, monocytes, macrophages, and mast cells, play roles in its pathogenesis [10,21,24,25]. These cellular interactions and immune mechanisms are illustrated in Figure 1.

Following activation by external or internal stimuli, dendritic cells in the epidermis produce various inflammatory cytokines, including TNF-α, IL-12, and IL-23, which promote the differentiation of naive T cells into Th1 and Th17 cells [2,14,19,29]. IL-17, IL-22, and IFN-γ cytokines produced by Th17 cells contribute to inflammatory processes, stimulating keratinocyte proliferation and neutrophil recruitment to the skin, thus exacerbating the inflammatory condition [2,8,27,29]. IFN-γ produced by Th1 cells also maintains the inflammatory cascade and skin barrier dysfunction [10,21,28]. Inflammatory cytokines result in an over-proliferation of keratinocytes, which causes exfoliation. This increases the production of inflammatory mediators (TNF-β, IL-1, IL-22, and IL-6), which generate a positive feedback loop that recruits more inflammatory cells to the skin, thus increasing the intensity of the inflammatory response [10]. It also enhances angiogenesis and aggravates psoriasis symptoms by increasing the production of vascular endothelial growth factor (VEGF) to serve the increased metabolic needs of rapidly proliferating skin cells [8,24]. In general, innate immune cells, especially neutrophils, are highly important in the early stages of psoriasis development, whereas T cell-dominated adaptive inflammation is a feature of stable plaques in the later stages.

Different signalling pathways are also essential in the maintenance and exacerbation of psoriasis. The NF-κB (TNFAIP3, NFKBIA, NFKBIZ) and JAK-STAT (STAT3) signalling pathways play a prominent role in the regulation of the inflammatory environment through excessive keratinocyte growth and immune cell activation [21]. The IL-23/IL-17 axis is substantial, as IL-23 maintains the activity of Th17 cells, which produce IL-17 and IL-22, promoting inflammation and keratinocyte proliferation [10]. In addition, adjacent keratinocytes, leukocytes, and fibroblasts also communicate to enhance and maintain inflammation [2]. In short, the interaction between immune cells, cytokines, and keratinocytes creates a self-sustaining chronic inflammatory cycle that leads to the persistent nature of the condition [8].

A deeper knowledge of the pathogenesis of psoriasis is essential for identifying new therapeutic targets for the development of more effective and targeted treatment strategies [4,10].

Future goals are further exploring the molecular mechanisms of psoriasis, gaining a better understanding of the role of genetic factors, and developing personalised treatments to improve the quality of life of the patients. The application of innovative therapies, such as nanotechnology-based drug delivery systems, could open up new possibilities for treatment. These new approaches may contribute to the long-term improvement of the condition of patients with psoriasis and provide an opportunity for more effective and long-term control of the disease [10,30,31,32].

## 6. Psoriasis Therapies and Their Limitations

The treatment of psoriasis involves multiple approaches, such topical, oral, biological, and intravenous therapies, as well as phototherapy [1,23]. As psoriasis is mainly a skin disease, topical treatment is the first choice in mild and moderate cases [23]. The majority of patients with psoriasis (80%) can be effectively treated with topical therapy [33]. However, these treatments present a number of challenges, including side effects, discomfort, and low patient adherence, which are significant problems for therapeutic success.

The currently available topical treatments are corticosteroids, vitamin D analogues (calcipotriene, calcitriol), retinoids (tazarotene), and calcineurin inhibitors (pimecrolimus, tacrolimus) [14,16,34]. These agents often have side effects, which limits their long-term use; the problems associated with each are summarised in Table 1 [5,34,35,36]. This situation creates an urgent need for well-tolerated topical therapies with minimal side effects that are suitable for long-term use [4,28].

Combination therapy can be more effective than monotherapy as it targets several pathomechanisms at one time. The combined use of corticosteroids and vitamin D analogues not only increases therapeutic efficacy but also moderates irritation. Similarly, the combination of topical retinoids and calcineurin inhibitors can boost treatment efficacy while reducing side effects [12,14,17]. Previous studies have also indicated that salicylic acid can improve the permeation of calcineurin inhibitors, vitamin D analogues, and corticosteroids through the skin, thereby helping to improve their therapeutic profile [37].

Moderate and severe psoriasis may require systemic therapies, which include methotrexate, cyclosporine, and acitretin [1,17,23]. Although great advances have been made in newer biologic therapies and phototherapy, they are not available or appropriate for all patients due to their high cost and potential immunological side effects [31]. Systemic drugs are very effective and can relieve the patient of symptoms for a period of time, but the risk of side effects is much higher than with topical treatments; this is presented in Table 2. Thus, in moderate to severe forms of the disease, the combination of systemic and topical agents could be necessary to achieve a complete cure [22].

The priorities of psoriasis treatment are to alleviate symptoms, clear the skin, and make patients’ daily life easier. The optimal therapy is selected on an individual basis, considering disease severity, plaque location, comorbidities, patient preferences, response to prior treatments, and expert opinion [9,27,31]. Maintaining patient adherence remains a major challenge in the management of psoriasis. The main reasons for discontinuing therapy include low efficacy, unsatisfactory cosmetic results, and suboptimal doctor–patient relationships [12,22]. In addition, conventional topical treatments are often uncomfortable, difficult to apply, or take too long to achieve the desired therapeutic effect [12,22,27]. More than 50% of patients are dissatisfied with the current treatment options, which results in poor patient compliance [27,38].

Although advances have been made in current therapeutic options, high patient dissatisfaction rates and low adherence suggest a potential need for a well-tolerated, non-steroidal topical therapy [12,33,39]. Novel nanocarrier-based strategies offer a promising alternative, as they improve the skin penetration of active ingredients, reduce side effects, and can provide effective therapy at lower doses, thus increasing patient satisfaction. The aim for the future is to develop therapeutic approaches that are not only more effective and safer, but also better adapted to patients’ lifestyles and expectations [17].

New topical therapeutic options include tapinarof nanogels, which may offer a promising alternative to current treatments as a non-corticosteroid agent. Tapinarof is an aryl hydrocarbon receptor (AhR) agonist that exerts anti-inflammatory and antioxidant effects, thereby helping to reduce plaque formation and inflammatory processes [33,40]. Clinical trials have demonstrated that tapinarof is effective in improving skin lesions and is well-tolerated in the long term, with a more favourable side effect profile than that of conventional corticosteroids [33]. Therefore, this compound could potentially be a new and promising option for the treatment of psoriasis [40].

**Table 1:** Topical treatments for psoriasis: mechanisms, side effects, and examples.

Therapy	Mechanism	Adverse Effects	Examples	References
Corticosteroids	Inhibit the production of cytokines and reduce inflammatory mediators	Tachyphylaxis, atrophy, stretch marks, erythema	Clobetasol, betametazon, mometazon	[9,12,22,23,33]
Vitamin D analogues	Inhibit dendritic cell maturation, T-cell activation, and keratinocyte proliferation	Skin irritation, burning, erythema	Calcipotriene, calcitriol	[2,12,17,23,25]
Retinoids	Inhibit keratinocyte proliferation	Erythema, peeling, skin irritation, burning, itching	Tazarotene, tretinoin	
Calcineurin inhibitors	Reduce T-cell activation and the production of inflammatory cytokines (IL-2) by inhibiting the enzyme calcineurin	Skin irritation, burning, itching	Tacrolimus, pimecrolimus	[22,23,27,33,41]
Keratolytics	Reduce the intercellular cohesion of the stratum corneum by dissolving the intercellular cementum	Frontal headache, central nervous system symptoms, metabolic acidosis, tinnitus, nausea, vomiting	Salicylic acid	[23,42]

**Table 2:** Systematic treatments for psoriasis: mechanisms, side effects, and examples.

Therapy	Mechanism	Adverse Effects	Examples	References
Dihydrofolate reductase inhibitors	Block the proliferation of keratinocytes and immune cells by inhibiting dihydrofolate reductase	Dry skin, hair loss, liver toxicity, risk of skin cancer, nausea, infections, bone marrow suppression	Methotrexate	
Retinoids	Inhibit keratinocyte proliferation	Nausea, hepatotoxicity, infections, xerosis, nail and hair fragmentation, teratogenicity	Acitretin	[1,9,17,23]
Calcineurin inhibitors	Reduce T-cell activation and the production of inflammatory cytokines (IL-2) by inhibiting the enzyme calcineurin	Dry skin, cardiovascular and gastrointestinal problems, gingival hyperplasia, tremor, leukopenia, hepatotoxicity, nephrotoxicity, hypertension, increased immunosuppression	Cyclosporine	[1,2,6,17,41]
Biological therapies	Inhibit cytokines or cytokine receptors (IL-12/23 inhibitors, TNF inhibitors, IL-17 inhibitors)	Expensive, safety concerns, high risk of malignant tumours and facial paralysis	Etanercept, adalimumab, ustekinumab, infliximab	[1,2,9,23]
Phototherapy	Causes cell death by apoptosis, necrosis or autophagy; reduces epidermal proliferation	Melanoma, photoaging, burning, erythema, pruritus, xerosis, pain, and discomfort	Ultraviolet B light, psoralen ultraviolet A light, photodynamic therapy	[1,2,6,23,41]

## 7. New Therapeutic Strategies for Psoriasis

Over the last decade, several new therapies have been developed to attack the physiological mechanisms behind inflammatory diseases. The US Food and Drug Administration (FDA) has approved multiple new agents for the treatment of psoriasis, which have led to the establishment of new therapeutic options. Notably, IL-17 and IL-23 antagonists, as well as oral TYK2 inhibitors and topical agents such as PDE4 inhibitors and AhR agonists, have shown significant clinical efficacy. Table 3 summarises recently FDA-approved agents, detailing their applications, drug classes, and indications [43,44,45,46].

## 8. Tapinarof

### 8.1. Structural Properties and Pharmacological Relevance of Tapinarof

The chemical properties of tapinarof are also crucial for its pharmacological application. It is a solid white powder with the chemical formula C_17_H_18_O_2_ and a molecular weight of 254.32 g/mol [76,77]. Its solubility in DMSO is 2 mg/ml, and it is insoluble in water, which is a challenging issue for drug technology development [78]. Stability studies have demonstrated that it degrades in aqueous environments, and the degradation process is enhanced at higher temperatures and with exposure to UVA radiation. Degradation of about 50% in the presence of molecular oxygen was observed in a pH 7.4 aqueous solution after 48 h at laboratory temperature [79].

**Figure 2:**
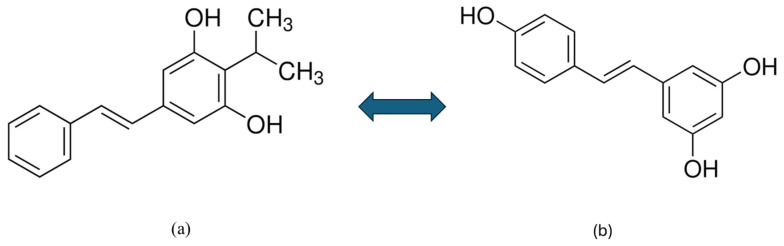
Chemical structure of tapinarof (**a**) and resveratrol (**b**) [77].

Tapinarof belongs to the group of polyphenols, specifically phenolic transstilbenes, which are produced by certain bacteria. It is structurally and functionally different from polyphenols of plant origin, such as resveratrol. However, tapinarof can be considered an isopropyl analogue of resveratrol, as shown in Figure 2. Tapinarof and its structurally related derivatives— resveratrol, pterostilbene, and pinocylvin—have been extensively studied [79,80]. Natural stilbenes are produced by many plants as a defence mechanism against various stresses, such as excessive UV radiation, heat stress, insect attack, and fungal or bacterial infections [79].

Although tapinarof and resveratrol are structurally similar compounds, their activities are significantly different [80]. Resveratrol is a natural phenolic compound produced by many plants (grapes, nuts, and berries) and acts as a partial agonist at the AhR. Research has shown that resveratrol is able to attenuate imikimod (IMQ)-induced psoriasis-like dermatitis in mice by reducing IL-17A and IL-19 [29]. While tapinarof directly activates the AhR pathway, resveratrol is only weakly or barely able to do so. The different origins (bacterial vs. plant) likely contribute to the differences in their activity. Profiling studies have demonstrated that the two compounds have different interactions with the AhR-ARNT complex, which is important for their biological effects. This suggests that tapinarof has a more specific and potent mechanism of action in activating AhR than resveratrol [80].

### 8.2. Tapinarof as a New Nonsteroidal AhR Modulator

Tapinarof (WBI-1001), also known as benvitimod or GSK2894512, is a non-steroidal anti-inflammatory compound that has a significant effect on the regulation of the immune response [4,10,16,81,82]. It is a novel topically applied agent that targets aryl hydrocarbon receptors, which are conductive to antioxidant activity, skin barrier protein expression, and cytokine regulation [11,12,83]. Tapinarof binds to AhRs to modulate the expression of skin barrier proteins, thereby reducing skin inflammation and promoting the recovery of skin barrier function. It also inhibits Th17 cell differentiation and reduces the expression of inflammatory cytokines such as IL-17, IL-22, and IL-23 by its mechanism of action. Furthermore, it alleviates oxidative stress through its antioxidant activity [11,83,84]. The role of AhR is crucial in this disease. Tapinarof and other AhR modulators could open up new perspectives in the treatment of psoriasis and other inflammatory skin diseases [16,83,85].

This compound was originally developed in China, and it has been evaluated for efficacy and safety in a comprehensive clinical trial programme. The formulation is optimised for twice-daily application and contains proprietary excipients to enhance its topical efficacy [11,16,86,87]. The FDA approved tapinarof 1% cream for the topical treatment of plaque psoriasis in adults in May 2022 [86,88,89].

It has shown excellent efficacy and favourable tolerability in clinical trials due to its unique mechanism [26,83,90]. It is also currently being investigated for the treatment of atopic dermatitis [33,83,91,92]. Whether marketed as benvitimod or tapinarof, despite formulation differences, this innovative molecule could become a promising alternative for the treatment of plaque psoriasis [85].

### 8.3. The Biological Origin of Tapinarof

Tapinarof is a natural, non-herbal polyphenol that is produced by Photorhabdus luminescens [13,81,93]. It is a bioluminescent, Gram-negative bacillus that lives in symbiosis with the nematode Heterorhabditis [26,33,82]. Nematodes harbour this bacterium in their intestinal tract and excrete it into insects. The bacterium produces metabolites that destroy the host, thus providing the optimal environment for nematode development. Observations showed that insects infected with nematodes did not decompose as quickly after death as those that were not exposed to the parasite [13,16].

The discovery of tapinarof was based on a random study of secondary metabolites of P. luminescens. The first observations date back to 1959, when Dutky and colleagues [94]. observed that insects infected with nematodes did not decompose rapidly, in contrast to the rapid decomposition observed in their absence [12,13]. This phenomenon suggested that P. luminescens may produce metabolites that have an antibiotic nature with anti-inflammatory properties, preventing rapid degradation of the infected host, and are responsible for the observed phenomenon [26,88].

Researchers isolated and identified the bioactive molecule produced by P. luminescens, called 3,5-dihydroxy-4-isopropylstilbene (also known as tapinarof) [16,90]. Subsequent research has characterised the compound in detail, which has been shown to produce immunomodulatory and anti-inflammatory effects when bound to AhR [16,81]. This led to the pharmaceutical development of the compound, which eventually became a topical therapy for the treatment of psoriasis and other skin diseases.

### 8.4. The Role of AhR Activation and the Therapeutic Potential of Tapinarof in the Treatment of Inflammatory Skin Diseases

The aryl hydrocarbon receptor is a cytoplasmic, ligand-dependent transcription factor that is activated by several exogenous and endogenous ligands [36,84,93]. It is widely expressed in various skin cells, including keratinocytes, fibroblasts, masocytes, and melanocytes, where it functions as a chemical sensor, converting external and internal stimuli into biological responses [14,36,89]. In healthy skin, AhR signalling is essential for maintaining skin homeostasis, regulating the immune response, and modulating inflammatory processes [36,84,93].

The expression of AhR is modified in psoriasis, influencing the pathogenesis of the disease. Activation of AhR regulates the terminal differentiation of CD4+ T-helper cells (Th17 and Th22) and the production of IL-17 and IL-22 cytokines, which are responsible for the maintenance of inflammation [22,26,33,80].

AhR can be activated by exogenous ligands such as environmental toxins (e.g., 2,3,7,8-tetrachlorodibenzo-p-dioxin) and endogenous ligands such as tryptophan derivatives [26,29,84,86]. After AhR binds to the ligand, the AhR–ligand complex migrates to the nucleus where it dimerizes with the AhR nuclear translocator (ARNT). The resulting AhR–ligand–ARNT complex binds to specific DNA recognition sites, triggering gene transcription and affecting skin barrier function, keratinocyte differentiation, and immune cell activity [26,29,87,93].

Since AhR activation is associated with the regulation of inflammatory and immunological processes in the skin, AhR agonists thus provide a promising therapeutic strategy for the treatment of inflammatory skin diseases [13,26,27,33,87]. In vitro and in vivo studies have demonstrated that AhR deficiency induces an enhanced inflammatory response, which increases the production of pro-inflammatory cytokines and makes the skin more vulnerable to inflammatory processes [16,29]. Furthermore, since keratinocyte proliferation and differentiation play a central role in the development of psoriasis, inhibiting keratinocyte hyperproliferation is also considered an effective therapeutic approach to treat the disease [36].

Through the activation of AhR, tapinarof induces anti-inflammatory and antioxidant effects, and repairs the skin and maintains its homeostasis (Table 4) [39,55,80,90]. It downregulates the production of inflammatory cytokines such as IL-17 and IL-22 [13,55,80]. These cytokines play a central role in keratinocyte proliferation, so their inhibition helps to suppress excessive cell division and restore normal skin structure. In this way, they do not only play a role in alleviating inflammatory symptoms, but also support the long-term health of the skin [29,33,90].

In addition, tapinarof upregulates the production of skin barrier proteins with reduced expression in psoriasis, such as filaggrin and loricrin, resulting in normalising of the skin barrier [55,84,88]. It also promotes the maintenance of skin function by regulating the gene expression of immune cells and skin cells [26,29,33,80].

Tapinarof also has antioxidant properties, partly due to its own chemical structure and partly due to activation of the nuclear factor erythroid 2-related factor 2 (Nrf2) pathway [35,81,88]. The 4′-OH group plays an significant role in the antioxidant activity of stilbenols [81]. In addition, the molecule contains two phenolic groups that directly neutralise reactive oxygen species (ROS): superoxide anions and hydroxyl radicals. By activating the AhR-Nrf2 transcription factor pathway, it promotes the expression of antioxidant enzymes such as NADPH quinone oxidoreductase 1 (NQO1) and heme oxygenase-1 (HO-1), decreasing oxidative stress. This dual effect may help to moderate psoriasis-associated cell damage [83,84,86,92].

Tapinarof also has antimicrobial activity, especially against Gram-positive bacteria and fungi, which can promote the balance and condition of the microbiome of the skin [90].

### 8.5. Results of Clinical Trials

Tapinarof is a first-in-class AhR approved by the FDA for the treatment of psoriasis, and it is currently being investigated for the treatment of atopic dermatitis as well [88,91,92,95]. It is presently only available as a cream. Its efficacy and safety have been evaluated in several preclinical and clinical trials in recent years, particularly in the treatment of psoriasis [12,28,33,55].

#### 8.5.1. Preclinical Studies

Preclinical studies of the mechanism of tapinarof have shown that its effects are strongly dependent on the presence of AhR and have confirmed its anti-inflammatory action, providing the basis for its clinical development [27,80].

Its effects were investigated in an IMQ-induced psoriasis mouse model. Topical application of 1% tapinarof cream alleviated psoriasis symptoms and reduced IL-17A expression in wild-type mice [96]. Tapinarof application also reduced pro-inflammatory cytokine levels and alleviated clinical signs of inflammation in mouse models of IMQ-induced psoriasis and lipopolysaccharide-induced ear oedema [27,29,80,86]. Its safety profile has also been investigated: a skin sensitisation test with 8% tapinarof cream in guinea pigs did not show significant skin irritation. Although human data on the risks of application are not available during lactation and pregnancy, animal studies have shown the presence of tapinarof in breast milk, but there is no evidence of teratogenicity [27].

Zhu et al. investigated the effect of tapinarof in two mouse models of psoriasis induced by imiquimod (IMQ) and interleukin-23 (IL-23). They observed that while tapinarof ameliorated IMQ-induced psoriasis-like dermatitis, there was also evidence of decreased keratinocyte proliferation, reduced epidermal thickness, and normalised differentiation. However, it paradoxically exacerbated inflammation in the IL-23-induced model. Tapinarof treatment in the IL-23 model led to increased epidermal thickness and the presence of differentiated epithelial dysplasia.

In contrast, Urashima et al. demonstrated that tapinarof suppressed IL-23-induced psoriasis-like dermatitis in mice, reducing ear thickness, inflammatory cell infiltration, and pro-inflammatory cytokine expression. A main difference between the two studies was in the timing of tapinarof administration. Zhu et al. applied tapinarof as a prophylactic treatment (concurrently with IL-23 injection), whereas Urashima et al. initiated treatment after inflammation had already been established. This temporal variation may explain the divergent results, suggesting that the immunomodulatory effects of tapinarof are context-dependent and may change according to the stage of the disease [14,97].

Overall, in the mouse models, tapinarof treatment attenuated keratinocyte proliferation and expression of inflammatory cytokines, leading to an alleviation of lesions characteristic of psoriasis. In addition, its antioxidant properties reduced the production of reactive oxygen species in keratinocytes, which further reduced the inflammation [29].

#### 8.5.2. Clinical Studies

In a phase II trial, 227 adult patients (aged 18 to 65 years) with plaque psoriasis were randomised to receive tapinarof cream at a concentration of 1% or 0.5% once or twice daily and were compared to the placebo group to assess efficacy and safety [12,27,33,83]. Treatment success was determined by PGA scores (Physician Global Assessment for Psoriasis) and PASI scores [27,29]. By the end of week 12, a higher proportion of those using 1% tapinarof (65% for twice daily and 56% for once daily) had achieved a “clear” or “almost clear” PGA score compared with the control group (11% for twice daily and 5% for once daily) [33,86,88]. The PASI-50 and PASI-75 response rates were also significantly higher in the tapinarof group (PASI-50: 71–92%, PASI-75: 46–65%) compared to the placebo group (PASI-50: 10–32%, PASI-75: 5–16%) [10,12,27,33].

Two major 12-week phase III trials (PSOARING 1 and PSOARING 2) included a total of 1025 adult patients (aged 18 to 75 years) with moderate to severe psoriasis [27,55,86]. The results confirmed previous observations that patients using tapinarof had improved PGA and PASI scores compared to the control group [10,16,55,86]. In these studies, once-daily application of 1% tapinarof cream significantly reduced PASI scores [10,16,33,90]. By the end of week 12, the PGA endpoint was reached by 35.4–40.2% of patients in the tapinarof group compared to only 6.0–6.3% in the control group [13,16,29]. The PASI-75 improvement rate was 36.1–47.6% in the tapinarof group compared to only 6.9–10.2% in the control group [27,55,88]. Overall, 85.8% of patients in the trials reported that tapinarof helped them to easily manage their psoriasis and 62.9% agreed that tapinarof cleared their skin and prevented recurrence [33,55].

#### 8.5.3. Long-Term Safety Studies

The PSOARING 3 study included 763 patients who had previously completed PSOARING 1 or 2 and were allowed to receive tapinarof for up to 52 weeks from baseline to the end of PSOARING 3 [12,55,86,91]. The aim of the 40-week phase was to evaluate long-term efficacy, safety, and ability to maintain the remission of tapinarof [12,27,33]. In this study, tapinarof 1% cream was applied in long-term, intermittent periods, and treatment provided a lasting effect for 12 weeks followed by a 4-week break [27,86,91]. A total of 40.9% of patients achieved complete skin clearance (PGA score = 0) at least once, and of those with active disease at baseline (PGA score ≥ 2), 58.2% achieved a PGA score = 0 on one or more occasions [12,27,86,88]. The results of the PSOARING 3 trial showed that tapinarof treatment not only produced durable improvement but also helped patients maintain symptom-free status for up to four months after the discontinuation of therapy. Longer-term clinical trials have shown low relapse rates with tapinarof, suggesting that tapinarof not only reduces symptoms but may also contribute to longer remission maintenance [12,27,88,91]. In addition, no tachyphylaxis was observed with prolonged use [13,27,91].

#### 8.5.4. Pharmacokinetics

Based on pharmacokinetic studies, both 1% and 2% tapinarof cream had low transdermal absorption [33]. Peak blood concentrations were observed on the first day of treatment and decreased significantly afterwards, which led to minimal systemic exposure. This is probably because the impaired skin barrier of psoriatic lesions at the start of therapy facilitates increased absorption, which may support early therapeutic efficacy. However, tapinarof activates the AhR during therapy, which boosts the expression of skin barrier proteins such as filaggrin, loricrin, and involucrin. As a consequence, the skin barrier is progressively restored over time, further reducing systemic absorption without decreasing local efficacy [27,33,88,98].

Therefore, the risk of systemic side effects remains low and no significant drug–drug interactions or cardiovascular effects have been reported [13,33]. The drug is mainly metabolised in the liver via CYP1A2 and CYP3A4 enzymes, while oxidation, sulfation, and glucuronidation occur in hepatocytes and the drug is strongly bound to plasma proteins (~99%) [27,33,86].

#### 8.5.5. Side Effects

During treatment, the most commonly reported side effect is folliculitis [82,88,91]. Contact dermatitis [16,28,86], upper respiratory tract infection [10,12,55], headache [10,16,88], and itching [27,55,88] have also been reported. Other less common adverse reactions are nasopharyngitis, influenza, urticaria, and drug-induced skin rash [27]. The majority of adverse reactions are mild to moderate in severity, and only a small proportion of patients discontinue treatment because of them [28,33,86,90].

Clinical studies have shown that VTAMA (tapinarof 1% cream) is generally well-tolerated in patients with psoriasis. Most adverse events have been mild to moderate in severity, and long-term use of the cream has not revealed any new safety risks [13,55,88].

Clinical trials are currently ongoing in the paediatric population (2–17 years), in which all participants receive 1% tapinarof cream once daily [99]. This is important because approximately 25% of psoriasis cases occur before the age of 18 years [27,90]. Trials have been carried out in atopic dermatitis, and the results show that tapinarof is safe for children over 2 years [100,101,102,103]. However, further research is needed to evaluate the safety of the product in pregnant patients [27,90].

Ghani et al. compared tapinarof with another new topical agent, roflumilast, in terms of safety and efficacy. Their findings showed that tapinarof was associated with more frequent but less severe side effects (folliculitis and contact dermatitis), while roflumilast showed less frequent but more severe side effects (diarrhoea, headache, and insomnia). However, further comparative clinical trials are needed to determine which agent is more effective and cost-effective in the long term [104].

However, further combination treatment options remain the subject of required trials. Research is ongoing to evaluate whether tapinarof in combination with other therapeutic modalities, such as biological agents or other topical agents, can enhance the efficacy and durability of treatment [12,33].

In parallel, studies are being carried out to assess the therapeutic equivalence of the tapinarof cream 1% developed by Teva Pharmaceuticals USA and the VTAMA tapinarof cream 1% already approved by Dermavant Sciences in adult patients with plaque psoriasis [105]. Separate studies have also investigated the efficacy and safety of the VTAMA (tapinarof) cream, 1% formulation, in patients with intertriginous psoriasis (underarm or groin area) [106].

Overall, the results of clinical trials have shown that the once-daily use of 1% tapinarof cream is safe and effective for the treatment of mild-to-moderate psoriasis for up to one year [13,91]. This cream’s favourable clinical profile makes it a promising therapeutic option, particularly for those who do not respond well to other treatments or who are seeking a long-term, sustainable, steroid-free therapy [16,28,33].

## 9. Nanotechnology in Dermatology

Nanotechnology is a scientific field that focuses on the development, synthesis, and characterisation of materials at the nanoscale (1–100 nm), creating new opportunities for drug delivery [8,17,107]. In recent decades, there has been a rising interest in nanotechnological solutions for dermal and transdermal therapies, particularly in the development of nanoparticle drug delivery systems, which have been playing a dominant role since the 1990s [31,108,109,110]. The application of nanoparticles in drug delivery provided valuable opportunities to enhance the therapeutic outcomes of different types of drugs, and may offer innovative strategies in the treatment of psoriasis [30,31].

One of the most important aims of pharmaceutical nanotechnology is the application of therapeutic and biocompatible drug carriers in nanoforms [111]. Nanocarriers provide the possibility for targeted delivery of active ingredients, which increases bioavailability and thereby enhances therapeutic efficacy [8,107,112].

Nanoparticles play a significant role in transdermal drug delivery, as their large surface area promotes skin penetration, retention, and sustained release [37,107,111]. Their efficacy is highly dependent on their penetration through the skin barrier, which is influenced by parameters such as particle size, molecular weight, surface charge, and pH [31,107,111]. Conventional drug formulations, such as creams and ointments, often have limited permeation, which makes it difficult to deliver drugs through the skin [9]. In contrast, nanotechnology can evade this obstacle by reducing particle size, which improves drug penetration through the stratum corneum, offering the possibility of targeted delivery of drugs to deeper layers of the skin [18,31,111].

Furthermore, drugs encapsulated in nanocarriers are protected from degradation, increasing the half-life of the active drug and minimising systemic toxicity [18,25,41]. In addition, their large surface area promotes the solubility of active ingredients and thus their bioavailability [31,112,113].

This is especially essential for hydrophobic drugs, as a significant proportion of novel drugs are lipophilic in nature and have poor water solubility, which limits their bioavailability and drug delivery efficacy [108]. Approximately 40% of drugs on the market and nearly 90% of molecules under development have poor water solubility, which is often a major cause of therapeutic failure [108,114,115]. Nanotechnology has revolutionised this field, as nanoparticle-based drug formulations can significantly improve the dissolution rate, permeation, and therapeutic efficacy of drugs [108,114]. This is advantageous in topical drug delivery, where the stratum corneum is the major limiting factor, acting as a primary barrier to drug entry [2,111,116]. Nanotechnology-based formulations offer an excellent opportunity for targeted and effective transdermal delivery of drugs with limited water solubility [108,112,114].

The active substances used to treat psoriasis (corticosteroids, retinoids, and immunomodulators) often have limited skin penetration and can cause severe side effects [8,111]. Nano-sized drug carriers offer the potential to address these issues [8,18].

## 10. Nanoparticle Carriers

In recent decades, several new generations of nanoparticle carriers have emerged that offer promising opportunities for topical therapies against psoriasis. Nanoparticle carriers can be grouped into four main categories: nanoparticles, nanofibres, physical carriers, and matrix nanocarriers [8,117]. In this section, we evaluate the advantages, limitations, and applicability of the most common nanoparticle types used in experimental psoriasis therapies.

### 10.1. Nanoparticles

#### 10.1.1. Vesicular Carriers

Liposomes, niosomes, transferosomes, and ethosomes are vesicular carriers [8,117].

Liposomes are nano-sized, phospholipid-based vesicular structures that form spontaneously in aqueous environments and are widely used as biocompatible, low-toxicity nanocarriers to enhance drug solubility, controlled release, and targeted skin delivery [8].

In a recent study, cyclosporin-loaded cationic liposomes developed by Walunj et al. achieved an entrapment efficiency of 93%, with a particle size of ~111 nm and zeta potential of +41 mV. In vivo application of the liposomal gel decreased psoriasis-related symptoms in an IMQ-induced mouse model. The treatment led to a 3.40-fold reduction in IL-22, a 1.47-fold reduction in IL-17, and a 1.71-fold reduction in TNF-α levels. Significant reductions were also observed in PASI scores, ear thickness, and spleen-to-body weight ratios, which confirm their therapeutic potential. Despite these advantages, challenges related to large-scale production, formulation reproducibility, and long-term physical stability hinder their broader clinical translation. Moreover, high drug loading can also lead to precipitation or particle aggregation, particularly when using thin-film hydration methods [118].

Niosomes are non-phospholipid-based bilayer vesicles composed of non-ionic surfactants and cholesterol, offering improved chemical stability and lower production costs compared to liposomes [17,117]. Their structural flexibility provides improved skin penetration [17].

A study by Abu Hashim et al. investigated an acitretin-loaded niosomal gel for topical psoriasis therapy. The optimised formulation achieved high drug entrapment (90.32 ± 3.80%), a particle size of 369.73 ± 45.45 nm, and a zeta potential of −36.33 ± 1.80 mV. Ex vivo skin permeation assays demonstrated a 3.16-fold increase in cumulative permeation and significantly enhanced drug deposition in the viable epidermis and dermis (330.86 ± 5.32 µg/cm^2^), compared to a conventional acitretin gel. In vivo, the formulation showed antipsoriatic efficacy in a mouse tail model, promoting orthokeratosis and reducing epidermal thickness, with no signs of skin irritation. Despite these promising findings, its clinical relevance remains uncertain due to the lack of human data. Furthermore, long-term stability and safety were not fully carried out, as the formulation was only tested under limited storage conditions and short-term application [119].

Transfersomes are vesicular particles consisting of ultradeformable lipid bilayers and at least one internal aqueous compartment. Their flexibility allows the vesicles to deform without structural damage and effectively penetrate the skin barrier, thereby facilitating targeted delivery of the drug to deeper layers. They provide a more efficient and controlled drug delivery than conventional liposomes [8,17].

Vohra et al. developed a nano-transferosomal gel loaded with aloe vera and vitamin E, which showed high entrapment efficiency (92.29%) and a stable zeta potential (−38.5 mV). The nanoformulations demonstrated notable anti-inflammatory effects and skin compatibility during topical application and significantly outperformed a commercial vitamin E cream. This was evidenced by results obtained in a TPA-induced mouse ear oedema model. However, the study lacked in vivo psoriasis-specific efficacy data, limiting its disease-specific relevance. Thus, while the carrier shows promise for herbal-based anti-inflammatory applications, its specific benefit in psoriasis therapy remains to be demonstrated in appropriate disease models [120].

Ethosomes are also ultradeformable phospholipid-based nanoparticles containing a high concentration of ethanol (20–45%) [8]. One of the major advantages of ethosomes over other lipid-based nanocarriers such as liposomes or transferosomes is that they are more efficient in penetrating the stratum corneum due to their ethanol content [8,41,117].

An ethosomal gel based on tacrolimus and hyaluronic acid was formulated by Dadwal and co-workers. The optimised nanoscale vesicles had high drug loading efficiency and excellent physicochemical properties. Ex vivo skin permeation studies using goat skin revealed a flux of 90.22 ± 0.52 µg/cm^2^/h for the ethosomal gel, compared to 72.15 ± 0.31 µg/cm^2^/h for the commercial tacrolimus formulation, resulting in an enhancement ratio of 1.33. These results indicate improved transdermal absorption and suggest that the combination of ethosomal nanocarriers with hyaluronic acid can offer a promising strategy for the topical treatment of psoriasis. However, further comparative preclinical and clinical studies are needed to confirm long-term safety and efficacy and to quantitatively assess the benefit over other nanocarriers [121].

#### 10.1.2. Lipid Nanoparticles

Examples of lipid nanoparticles include solid lipid nanoparticles (SLNs) and nanostructured lipid carriers (NLCs).

Solid lipid nanoparticles are spherical, lipid-based nanoparticles that stay solid at room and body temperature [17,117]. One of their main advantages over conventional liposomes is their more efficient drug delivery, which includes higher drug loading capacity, controlled release, and enhanced stability of the active substances [17,117].

In their study, Serini and co-workers reported that solid lipid nanoparticles containing curcumin and α-linolenic acid were able to reduce IMQ-induced inflammation in an in vitro psoriasis model, which was evidenced by decreases of 43% in IL-23, 73.7% in IL-6, and 26.5% in IL-8 expression in macrophages. In addition, the levels of two markers of ferroptosis (TFRC and MDA) were significantly reduced, suggesting an antioxidant effect. However, while these findings indicate that curcumin and α-linolenic acid-loaded SLNs may represent a promising nanocarrier-based strategy, the effects have so far only been demonstrated in vitro and further preclinical and clinical studies are needed to assess the translational potential [122].

Nanostructured lipid carriers contain physiologically compatible lipids, surfactants, and emulsifying agents. They can enhance skin hydration, strengthen the skin barrier, improve bioavailability, and provide targeted drug delivery. NLCs are particularly advantageous in the field of drug delivery systems due to their ease of production, biocompatibility, non-toxicity, and scalability [8].

For the treatment of hyperproliferative skin disorders, Llorente et al. developed riluzole-containing nanostructured lipid carriers. The optimised formulation demonstrated a mean particle size under 200 nm, a high entrapment efficiency (~87%), and a zeta potential around −25 mV. In vitro studies using HaCaT cells demonstrated that the formulation significantly inhibited cell proliferation in a dose-dependent manner (*p* < 0.0001), with effects comparable to free riluzole. In animal models, topical application of the preparation led to reduced skin thickness and visible alleviation of inflammatory signs. Although the study demonstrated promising physicochemical properties and therapeutic effects of riluzole-loaded NLCs, it did not include comprehensive in vivo toxicity data. Additionally, the formulation process is relatively complex due to the low solubility and light sensitivity of the active ingredient, requiring optimisation for stability and efficacy [123].

### 10.2. Nanofibres

Nanofibres are fibres or fibre-like structures made from natural or synthetic polymers that provide fluid absorption, moisture control, and gas permeability [117]. Nanofibres have an excellent surface-to-volume ratio, which allows for the efficient delivery of both hydrophilic and hydrophobic drugs [124]. They are promising in the treatment of psoriasis, where they are often used in combination with nanoparticles to promote synergistic drug release and skin regeneration [117].

Kang and coworkers investigated curcumin-filled nanofibre films using electrostatic fibre pulling. The optimised film contained curcumin at 58.7 ± 10.1 μg/cm^2^ with an entrapment efficiency of 56.5 ± 9.7% and tensile strength of 4.86 ± 0.14 MPa. In vitro skin deposition studies using a psoriasis mouse model showed a >2.0-fold increase in curcumin deposition with lipid-hybridised films compared to lipid-free controls. In vivo, treatment with the hybrid film significantly reduced skin thickening, scaling, and pro-inflammatory cytokines (TNF-α and IL-6), with anti-inflammatory efficacy approaching that of a commercial corticosteroid. These advantages suggest strong potential in psoriasis therapy. However, challenges such as curcumin-induced skin staining and limited dermal penetration highlight the necessity for further optimisation [125].

### 10.3. Physical Carriers

These systems are hybrids of hypodermic needles and transdermal patches containing hundreds of tiny, microscale needles, which create microscopic channels through the stratum corneum, enhancing drug penetration efficiency [117,126].

Du et al. designed a microneedle patch containing hyaluronic acid and methotrexate for the topical treatment of psoriasis. In vitro studies showed that the preparation preserved the antiproliferative activity of API against HaCaT keratinocytes and achieved ~90% drug release within 1 h. In vivo, the microneedles successfully penetrated both normal and psoriatic mouse skin (~150 μm), dissolved within 10 min, and significantly reduced epidermal thickness, ear swelling, and cytokine expression (IL-17, IL-23, Ki67) in an imiquimod-induced psoriasis model. Compared to oral methotrexate at the same dose (13.8 μg), microneedle-delivered methotrexate achieved superior therapeutic outcomes with less systemic toxicity. However, disadvantages include reduced mechanical strength at higher drug loads and the lack of long-term safety evaluation [126].

### 10.4. Matrix Nanocarriers

Matrix-based nanocarriers such as nanoemulsions and nanogels offer promising advantages in transdermal drug delivery. Nanoemulsions are kinetically stable dispersions of immiscible liquids (typically oil and water) with droplet sizes between 10 and 200 nm, which enhances drug solubility and bioavailability [112,113,115,116]. However, their low viscosity makes their application problematic, which can be solved using nanogels [115,127]. By incorporating nanoemulsions into a gel matrix, nanogels combine the advantages of gel formulations and nanotechnology to provide a more efficient drug delivery system [115].

Nanogels are a three-dimensional, nanoscale (20 to 250 nm) network of hydrophilic polymers with viscoelastic properties [109,111,128]. They are made from polymers or through heterogeneous polymerisation of monomers, and their structure can be stabilised by cross-linkages, either physical or chemical [3,8].

Their constitution affects their biocompatibility and biodegradation, which minimises toxicity and immune response. They are able to provide stable delivery and enhanced skin penetration of different types of drugs, provide protection against degradation and environmental effects, and also increase the stability and bioavailability of drugs [25,109,117,129]. Due to their high water retention capacity, they hydrate the skin surface while providing controlled and prolonged drug release, maintaining the desired therapeutic effect over a longer period of time [8,23,117,128].

Since psoriasis treatment requires long-term commitment, patient adherence is essential [3,8]. Nanogels support this by enabling sustained drug release, reducing dosing frequency, and improving treatment comfort and compliance [8,25,41,111].

Previous studies have confirmed that these new nanoformulations may present exciting potential not only for the treatment of psoriasis but also for other skin conditions.

Chandrashekhar and co-workers evaluated a tretinoin nanogel (0.025%) versus a conventional gel in a randomised, multicentre clinical trial involving 207 acne patients. The nanogel group showed significantly higher reductions in total (72.9% vs. 65.0%; *p* = 0.03) and inflammatory lesions (78.1% vs. 66.9%; *p* = 0.02), and fewer local adverse events (13.3% vs. 24.7%; *p* = 0.04) compared to the conventional formulation [130].

Avasatthi et al. developed a methotrexate-loaded nanostructured lipid carrier nanogel that achieved 47.32% drug release at 48 h, compared to 94.23% from conventional methotrexate gel, indicating sustained release. In a psoriasis mouse model, the preparation reduced PASI scores and restored skin histology, while the conventional gel resulted in persistent parakeratosis [131].

Kakade et al. formulated a tacrolimus-loaded nanogel based on nanostructured lipid carriers, which demonstrated over 90% sustained drug release over 24 h, excellent spreadability, and a high drug content of 99.73 ± 1.4%. In vivo, the nanogel improved skin elasticity, resolved psoriatic lesions in oxazolone and imiquimod models, and showed no cytotoxicity or irritation, supporting its effectiveness and safety [132].

Despite the remarkable advantages, their production faces several technological challenges, especially in high-volume manufacturing where reproducibility, stability, and cost-effectiveness are main factors [110,129,133].

Their stability is a critical factor, as they are sensitive to storage conditions such as temperature fluctuations and changes in humidity [110,112,133]. In addition, the desired quality cannot always be ensured in high-volume production [42,110,112].

**Figure 3:**
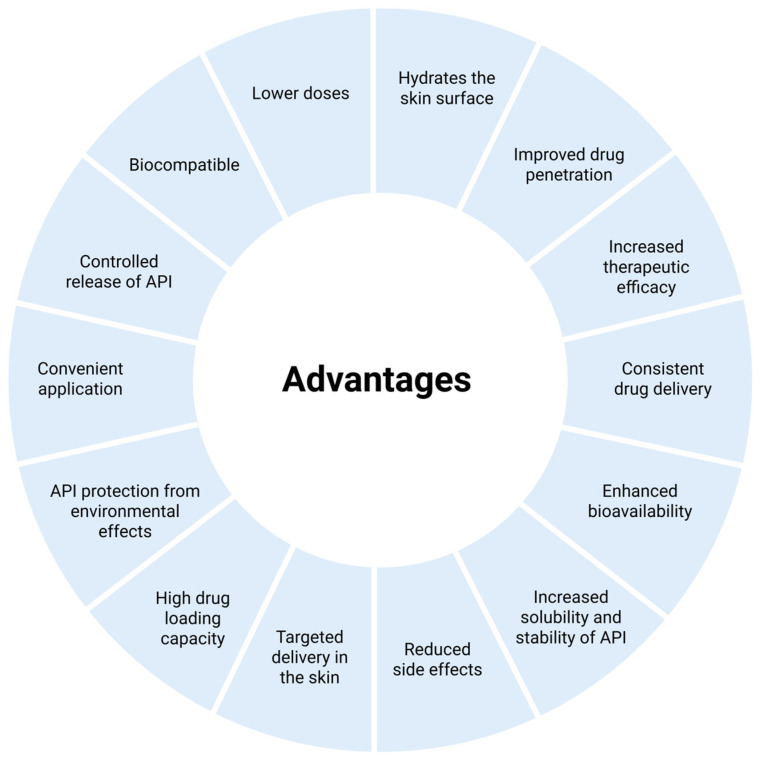
Advantages of nanogels in transdermal drug delivery (created with BioRender.com).

**Figure 4:**
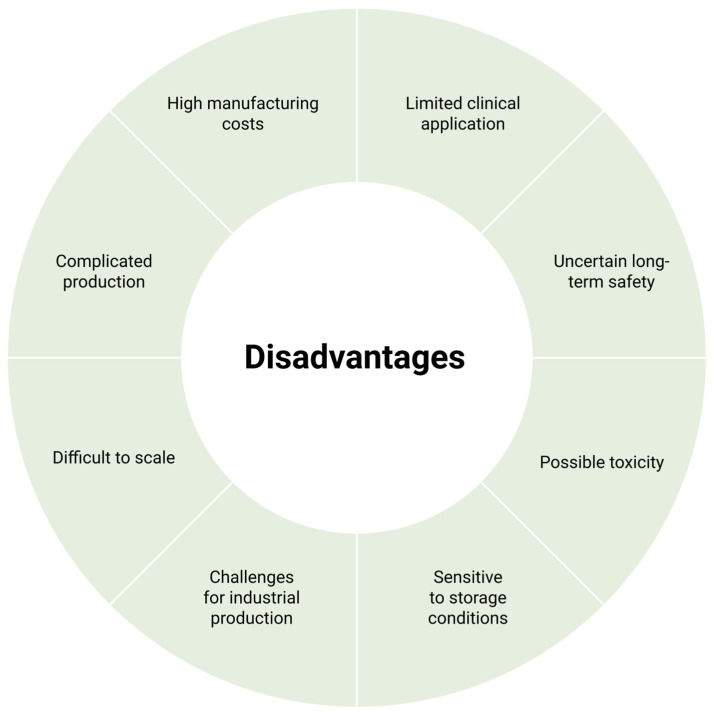
Disadvantages of nanogels in transdermal drug delivery (created with BioRender.com).

As these materials are gradually degraded in the body, long-term biocompatibility testing is essential as it is important that they do not cause any toxic side effects [41,133]. In light of this, nanogels have noticeably more advantages than disadvantages, as presented in Figure 3 and Figure 4 [3].

Nevertheless, nanogels represent one of the most promising developments in the field of drug delivery systems and are expected to play a prominent role in the treatment of advanced drug formulations such as psoriasis and other skin diseases in the future [134].

## 11. Tapinarof Delivery Systems

### 11.1. Patented Tapinarof Formulations

Currently, the only approved and commercially available formulation of tapinarof is VTAMA (tapinarof cream 1%), which was developed and patented by Dermavant Sciences [13,55,88]. This formulation is protected by a series of patents (e.g., US11590088B2, US11938099B2) covering its composition and use for chronic dermatological conditions such as plaque psoriasis [135]. and atopic dermatitis [136]. Other formulations exist at the research level or as patent registrations but not for clinical use. A US patent (US20210000758A1) describes the encapsulation of tapinarof in nanoparticles (e.g., nanomicelles, nanospheres) for ophthalmic applications such as the treatment of uveitis or macular degeneration [137]. Another patent (US20220160650A1) describes various tapinarof-based gel, ointment, and foam formulations designed to improve the stability and skin absorption of the active ingredient [138].

### 11.2. Challenges in Formulating Tapinarof

One of the major challenges in the dermatological application of tapinarof is its hydrophobic nature and instability, which make it difficult to formulate in conventional systems [139]. The compound is highly sensitive to temperature and humidity, which can negatively impact long-term stability and reduce therapeutic efficacy during storage [79,110,112,133].

Nanogels offer a promising strategy to overcome these challenges, as they are able to encapsulate the active ingredient, thereby protecting it from environmental degradation and improving long-term shelf-life [18,25,41]. Non-ionic surfactants and natural polymers not only support the solubilisation of the drug, but also improve biocompatibility and skin tolerability [10,24].

Furthermore, considering the chronic and relapsing nature of psoriasis, formulations should be designed for safe, prolonged use without causing irritation or sensitisation [10,20]. Patient adherence can be further improved by ensuring favourable application properties (e.g., texture, spreadability) and reducing the dosing frequency. Altogether, these factors highlight the urgent need for effective, steroid-free, and well-tolerated topical therapies that are suitable for long-term monotherapy or combination therapy without the risk of drug–drug interactions [28,33,55].

### 11.3. Nanogel-Based Tapinarof Formulations

To overcome formulation-related challenges of tapinarof, Balogh et al. developed nanogel-based delivery systems aiming to improve drug solubility, skin penetration, and stability. The nanogels were prepared using Carbopol 940 and 936 polymers combined with excipients such as tween 80, kolliphor, and oleic acid to enhance therapeutic performance. Dynamic light scattering (DLS) confirmed nano-sized particle distributions (151–173 nm), and rheological analyses demonstrated pseudoplastic behaviour with temperature-dependent viscosity, which is very important for spreadability and controlled release at skin temperature. During texture analysis, the formulations exhibited low compressive resistance, ensuring easy topical application. Cytotoxicity was evaluated on HaCaT keratinocyte cells via MTT assays. None of the tested formulations showed cytotoxicity. Notably, nanogel II showed a better release profile in the Franz diffusion assays, as 81% of tapinarof was released after 5 h compared to 52% for nanogel I, with a corresponding increase in drug flow. A wound healing study showed that the incorporation of tapinarof into the nanoformulations enhanced its antiproliferative and antimigratory activity. Taken together, these findings indicate that nanogel-based tapinarof delivery systems are a promising candidate for topical psoriasis therapy [139].

### 11.4. Future Directions

Preliminary in vitro studies on tapinarof-loaded nanogels have demonstrated promising results in terms of active ingredient absorption and cellular tolerability, indicating their potential as a novel topical delivery system [139]. However, these findings represent only an early stage of development. To support clinical application, further preclinical and clinical studies are essential to confirm the efficacy and safety of these formulations in vivo.

Preclinical studies in animal models (e.g., IMQ-induced mouse model) can provide information regarding dosing, pharmacokinetics, pharmacodynamics, and long-term stability.

Following successful preclinical testing, phase I clinical trials should be conducted in healthy volunteers to assess safety, local tolerability, and pharmacokinetics. If these results are appropriate, phase II trials involving psoriasis patients can evaluate efficacy and dose optimisation. Following these, phase III randomised, double-blind, placebo-controlled studies are required to confirm therapeutic benefit, long-term safety, and patient adherence. Although this is a lengthy process, it is necessary to make tapinarof nanogels a reliable and effective treatment option for patients with psoriasis.

During development, it is also essential to compare tapinarof nanogels with existing psoriasis treatment modalities, for example, steroids or other topical therapies. These comparative studies may reveal the benefits and potential limitations of nanogels, helping to determine their clinical applicability more accurately [12].

Additionally, efforts must focus on establishing reproducible, cost-effective manufacturing processes, as well as ensuring long-term stability and wide applicability. Addressing these challenges is essential to support the clinical integration of tapinarof nanogels as a safe, patient-friendly alternative for long-term topical management of chronic plaque psoriasis [28,39].

## 12. Conclusions

The complex and multifactorial pathophysiology of psoriasis continues to make it difficult to develop truly optimal treatments. Despite major advances in the use of systemic biologic agents for the treatment of moderate to severe forms of the disease, a large proportion of psoriasis cases remain untreated, mainly due to side effects of systemic drug treatments or inappropriate drug delivery through the stratum corneum in topical treatments. In this context, nanotechnology offers a revolutionary solution, particularly nanogel formulations that enhance drug penetration and bioavailability at the place of action. With proven potency in the treatment of psoriasis, incorporating tapinarof into nanogels can significantly improve therapeutic outcomes by targeting the skin more effectively and reducing potential side effects. Nanogels can also provide a controlled sustained release of the active ingredient, enhancing the stability and efficacy of tapinarof. This approach can enhance both the convenience and long-term efficacy of topical therapies. Additionally, integrating nanotechnology into psoriasis management could help tailor therapies to the individual needs of patients, improving their adherence to treatment and overall quality of life. Eventually, the incorporation of tapinarof nanogels into clinical practice could revolutionise the way psoriasis is treated, offering a patient-friendly approach to treating this challenging dermatological condition. Future studies should further explore tapinarof-loaded nanogel formulations to support their clinical translation and ensure safety, efficacy, and long-term therapeutic benefits in patients with psoriasis.

## Figures and Tables

**Table 3:** New active agents recently approved by the FDA.

Active Substance	Market Name	Application	Drug Class	Indication	FDA Approval Year	Ref.
Bimekizumab	Bimzelx	subcutaneous injection	IL-17A, IL-17F antagonist	moderate-to-severe plaque psoriasis	2023	[47,48,49]
Deucravacitinib	Sotyktu	oral application	TYK2 inhibitor	moderate-to-severe plaque psoriasis	2022	[43,47,48,49,50]
Roflumilast	Zoryve	topical application	PDE4 inhibitor	mild-to-severe plaque psoriasis	2022	[51,52,53,54]
Tapinarof	Vtama	topical application	AhR agonist	mild-to-severe plaque psoriasis	2022	[27,42,55]
Risankizumab	Skyrizi	subcutaneous injection	IL-23 antagonist	moderate-to-severe plaque psoriasis	2019	[43,44,56,57]
Certolizumab pegol	Cimzia	subcutaneous injection	TNF-α blocker	moderate-to-severe plaque psoriasis	2018	[58,59,60,61]
Tildrakizumab	Ilumya	subcutaneous injection	IL-23 antagonist	moderate-to-severe plaque psoriasis	2018	[62,63,64,65]
Guselkumab	Tremfya	subcutaneous injection	IL-23 antagonist	moderate-to-severe plaque psoriasis	2017	[43,66,67,68,69]
Brodalumab	Siliq	subcutaneous injection	IL-17A antagonist	moderate-to-severe plaque psoriasis	2017	[43,70,71,72]
Ixekizumab	Taltz	subcutaneous injection	IL-17A antagonist	moderate-to-severe plaque psoriasis, psoriatic arthritis	2016	[43,73,74,75]

**Table 4:** Molecular pathways of tapinarof [13,27,92].

Effect	Mechanism
Anti-inflammatory effect	Reduces the expression of Th2 cytokines (IL-4, IL-5, IL-13, IL-31) and Th17 cytokines (IL-17A, IL-17F), which are involved in the pathogenesis of psoriasis.
Skin barrier enhancement	Increases the expression of skin barrier proteins (filaggrin, loricrin, involucrin) and ceramide skin lipids, helping to normalise the skin barrier.
Antioxidant effect	Activates the Nrf2 pathway and reduces reactive oxygen species levels, contributing to the reduction in oxidative stress and the antioxidant response of the skin.

## Data Availability

Not applicable.
